# The Effects of Fabrication Conditions on the Construction of Zein–Curdlan Composite Nanoparticles and Their Emulsifying Attributes

**DOI:** 10.3390/foods15183278

**Published:** 2026-09-17

**Authors:** Chao Wu, Shijia Li, Xue Bai, Xiaojing Kang, Ran Wang, Mingkun Liu, Beibei Dou, Yang Liu, Han Chen

**Affiliations:** 1 Food Laboratory of Zhongyuan, Department of Nutrition and Health, China Agricultural University, Luohe 462000, China; wuchao980103@163.com (C.W.); lishijia@cau.edu.cn (S.L.); 15663112002@163.com (X.B.); tn2020036@zyfoodlab.com (X.K.); wangran@cau.edu.cn (R.W.); liumingkun0203@163.com.com (M.L.); doubeibei@zyfoodlab.cn (B.D.); liuyang@zyfoodlab.cn (Y.L.); 2 Key Laboratory of Functional Dairy, Co-Constructed by Ministry of Education and Bejing Government, China Agricultural University, Beijing 100193, China; 3 Engineering Research Center of Bio-Process, Ministry of Education, School of Food and Biological Engineering, Hefei University of Technology, Hefei 230601, China

**Keywords:** zein, curdlan, nanoparticles, Pickering emulsions, emulsion stability

## Abstract

In this study, zein–curdlan (CU) composite nanoparticles were fabricated via an anti-solvent precipitation method. The effects of preparation parameters, including the mass ratio, reaction pH, and temperature, on the physicochemical properties and emulsifying attributes of the composite particles were systematically investigated. Fourier-transform infrared (FTIR) and X-ray diffraction (XRD) analyses revealed changes in the local molecular environment and molecular organization following zein–CU association, while particle size, zeta potential, and surface hydrophobicity measurements further demonstrated fabrication-dependent changes in the physicochemical characteristics of the composite particles. As the CU ratio increased, the zeta potential decreased to −25.5 mV, accompanied by a reduction in surface hydrophobicity, suggesting a more hydrophilic CU-associated particle surface. At pH 8, the particles showed the smallest size among the pH series (244.6 nm), which may be related to enhanced zein deprotonation and interparticle electrostatic repulsion under the tested conditions. A preparation temperature of 40 °C produced the smallest particles and the most favorable emulsifying properties among the temperatures examined. Overall, this work provides evidence for the formation and emulsifying performance of zein/CU composite particles, broadening the application of plant protein–polysaccharide complexes in food-grade Pickering emulsion systems.

## 1. Introduction

Distinct from conventional emulsions stabilized by synthetic surfactants, Pickering emulsions are stabilized by solid particles, offering prominent advantages such as minimized emulsifier dosage, cost-effectiveness, and environmental friendliness [1]. Biopolymers, particularly proteins and polysaccharides, have been widely utilized as food-grade Pickering stabilizers owing to their abundance, biodegradability, and biocompatibility [2]. These particulate stabilizers undergo irreversible adsorption at the oil–water interface, forming a rigid protective barrier around the droplets that effectively suppresses coalescence through steric hindrance and imparts robust resistance against Ostwald ripening [3]. In recent years, driven by growing sustainability imperatives and expanding vegan consumer demands, plant proteins have garnered widespread attention for formulating food-grade Pickering emulsions, owing to their eco-friendly nature and excellent emulsifying properties.

As the primary storage protein in corn, zein possesses unique physicochemical properties, including pronounced hydrophobicity, a tunable secondary structure, and excellent self-assembly characteristics, making it highly promising for food applications [4]. The distinct solubility of zein endows it with a remarkable self-assembly capacity; particularly under shifting environmental polarities, it can be fabricated into micro-/nano-scale lipid carriers via the anti-solvent precipitation method [5]. The secondary structure of zein is highly sensitive to environmental conditions; an increase in water content within an aqueous ethanol solution alters solvent polarity, driving a conformational transition from an α-helix to a β-sheet. This transition is accompanied by the reorganization of intermolecular hydrogen bonds, the formation of a hydrophobic core, and enhanced molecular flexibility [4,6]. Leveraging its amphiphilic nature, zein can adsorb at the oil–water interface to serve as a Pickering stabilizer, establishing a dense physical barrier that effectively suppresses droplet coalescence and phase separation [7].

However, when utilized alone as a structural food component, pure zein particles frequently suffer from severe aggregation and poor dispersibility. These limitations restrict their efficacy in stabilizing Pickering emulsions, necessitating further modification to satisfy emulsification requirements [8]. Although physical and chemical modifications can reorganize protein structures and alter microstructures to improve emulsifying properties [9,10], they often incur high processing costs and fail to align with clean-label requirements. Intriguingly, compared to single plant proteins, composite particles fabricated via protein–polysaccharide self-assembly can precisely modulate interfacial behavior through amphiphilic complementarity. This approach effectively prevents droplet coalescence via the synergistic effects of steric hindrance and electrostatic repulsion, thereby substantially enhancing Pickering emulsion stability [11,12].

As an amphiphilic molecule, the flexibility of zein is closely associated with the incorporation of hydrophilic and lipophilic agents; generally, lipophilic substances tend to bind to the hydrophobic domains of zein, thereby altering its internal particle density and surface charge, whereas hydrophilic entities contribute to enhancing the colloidal stability of zein particles [13]. Curdlan (CU), a microbial polysaccharide celebrated for its unique thermal gelation, structural evolution, and biocompatibility, has attracted considerable interest in food matrix modification. Highly responsive to pH and temperature variations, CU exhibits multi-stage conformational transitions at the molecular level. In ambient aqueous solutions, CU predominantly exists as a triple-helix structure. Accumulating evidence indicates that alkaline environments or thermal induction can drive a self-assembly process, transforming the CU molecular chains from a triple helix into uncoiled single strands and ultimately into random aggregates [14]. This structural evolution endows CU with distinctive thermogelling behaviors: heating to approximately 55 °C followed by cooling yields a thermoreversible thermotolerant low-set gel maintained by weak hydrogen bonds; conversely, heating above 80 °C triggers intense hydrophobic association and extensive interchain entanglement upon cooling, culminating in a resilient, thermoirreversible high-set gel. Crucially, this unique polymeric network can directly cooperate with proteins via hydrogen bonding and hydrophobic interactions to establish more compact and homogeneous hybrid networks [15,16]. This transition—spanning ordered-to-disordered states and subsequent network restructuring—suggests that CU holds great potential to undergo profound interfacial synergy with hydrophobic proteins like zein, thereby precisely modulating their surface properties and colloidal behavior.

In this study, zein–CU composite nanoparticles were fabricated via anti-solvent precipitation. By comprehensively evaluating their particle size, zeta potential, microstructure, and FTIR profiles, we investigated how the zein-to-CU mass ratio, reaction pH, and temperature affected the physicochemical and emulsifying properties of the composite particles. Furthermore, their three-phase contact angles and interfacial properties were characterized to elucidate their capability to stabilize Pickering emulsions as particulate emulsifiers. By clarifying the assembly mechanism and emulsifying capacity of zein–CU composite particles, this study extends the application scope of plant protein–polysaccharide composite particles in food-grade Pickering emulsion systems, providing a theoretical basis for further developing functional foods based on Pickering emulsion systems.

## 2. Materials and Methods

### 2.1. Materials

8-Phenyl-1-naphthalenesulfonic acid (ANS) and zein were purchased from Sigma-Aldrich (Beijing, China); curdlan was purchased from Haios Biotechnology Co., Ltd. (Zibo, China); anhydrous ethanol was purchased from McLean Biochemical Technology Co., Ltd. (Shanghai, China); sodium dodecyl sulfate (SDS), NaOH and HCl were purchased from Sinopharm Chemical Reagent Co., Ltd. (Shanghai, China); trisodium phosphate was purchased from Jindiya Mei Technology Co., Ltd. (Shifang, China); phosphate-buffered saline (PBS), BCA assay kit, and bovine serum albumin (BSA) were purchased from Solarbio (Beijing, China); and deionized water was prepared in the laboratory.

### 2.2. Preparation of Zein–CU Composite Particles

Zein nanoparticles were prepared via an anti-solvent precipitation method with minor modifications to the protocol described by Zhong et al. [17]. Briefly, 2.0 g of zein was dissolved in 100 mL of a 70% (*v*/*v*) ethanol–water aqueous solution under continuous stirring overnight. This solution was then introduced dropwise into 100 mL of deionized water and stirred at 800 rpm for 1 h. Subsequently, half of the solvent volume was eliminated using a rotary evaporator (RE-5000, Baer Instrument and Equipment Co., Ltd., Nantong, China) at 46 °C for 20 min to yield a 2 wt% stock zein dispersion. In parallel, 2.0 g of curdlan was dispersed and hydrated in 200 mL of deionized water adjusted to pH 8.0 under stirring at 60 °C for 2 h.

Preparation of zein–CU composite particles (ZCPs): For the temperature series, the hydrated CU dispersion was maintained at 20 °C, 30 °C, 40 °C, 50 °C, or 60 °C, while zein:CU and pH were fixed at 1:3 and 6.0, respectively. For the mass ratio series, zein dispersion was slowly added to the hydrated CU dispersion at zein:CU ratios of 3:1, 2:1, 1:1, 1:2, and 1:3, while temperature and pH were fixed at 40 °C and 6.0, respectively. For the pH series, the composite dispersions were adjusted to pH 5.0, 6.0, 7.0, 8.0, or 9.0 using 0.1 M HCl and trisodium phosphate solutions, while temperature and zein:CU were fixed at 40 °C and 1:3, respectively. The mixtures were stirred at 800 rpm for 1 h to obtain zein–CU composite particle dispersions. Because the mass ratio and pH series may also change protein concentration and the ionic environment, respectively, the effects discussed below should be interpreted as condition-dependent trends within each one-factor series.

### 2.3. Particle Size and Zeta Potential Measurements

The average particle size, polydispersity index (PDI), and zeta potential of the pristine zein nanoparticles and ZCPs were determined using a Zetasizer Nano-ZS90 (Malvern Instruments Ltd., Malvern, UK) with minor modifications to the method of Wu et al. [18]. Prior to analysis, all samples were diluted 10-fold with deionized water to alleviate multiple scattering effects. The refractive indices of the protein and the dispersant (deionized water) were set at 1.450 and 1.330, respectively. All measurements were conducted at ambient temperature (25 ± 1 °C), and each sample was analyzed in triplicate to ensure reproducibility.

### 2.4. Fourier Transform Infrared Spectroscopy (FTIR)

Following the method described by Zeng et al. [19] with minor modifications, the freeze-dried granular powder was mixed with potassium bromide in a 1:100 ratio, ground, and pressed into tablets. FTIR spectra were recorded in transmission mode using KBr pellets on a Fourier transform infrared spectrometer (Nicolet iS5, Thermo Fisher Scientific, Waltham, MA, USA) used to scan the range from 4000 to 400 cm^−1^, with a resolution of 4 cm^−1^, and each sample was scanned 64 times.

### 2.5. Surface Hydrophobicity

Following the method described by Chen et al. [20] with minor modifications, 8-phenyl-1-naphthalenesulfonic acid (ANS) was used as a fluorescent probe to determine the surface hydrophobicity of protein microgel particles. ZCP solutions were diluted with PBS buffer (pH 7.0, 10 mmol/L) to concentrations of 0.1, 0.2, 0.3, 0.4, and 0.5 mg/mL. An 8.0 mmol/L ANS stock solution was prepared. Five microliters of the 8.0 mmol/L ANS solution was added to 1 mL of the diluted protein solution and mixed thoroughly. Incubation was conducted in the dark for 10 min. Fluorescence intensity was measured using an F-7000 fluorescence spectrometer (Hitachi, Tokyo, Japan) at an excitation wavelength of 390 nm, an emission wavelength of 470 nm, and a slit width of 5 nm. Surface hydrophobicity is expressed as the initial slope of the fluorescence intensity versus protein concentration curve.

### 2.6. X-Ray Diffraction (XRD)

The crystallinity of the particles was measured using an X-ray diffractometer (Bruker D8, Karlsruhe, Germany) according to the method described by Zeng et al. [19]: Solid powder samples were spread evenly on a glass slide at 25 °C. The measurement parameters used were as follows: a copper anode for Cu Kα X-rays; an acceleration voltage of 40 kV; and a tube current of 40 mA. The 2θ angle range was scanned from 5° to 40°, with a scan interval of 5 s and a scan rate of 0.1°/s.

### 2.7. Scanning Electron Microscopy (SEM)

Following the method described by Wu et al. [21] with minor modifications, a scanning electron microscope (SU9000, Hitachi, Tokyo, Japan) was used to observe the microstructure of the particles. The sample was diluted 10-fold with deionized water. A 5 μL drop of the sample was placed on a silicon wafer and dried in an oven at 35 °C. The silicon wafer with the sample was then mounted on the sample stage. Finally, gold was sputtered onto the sample using a sputtering system, and observations were made at 30,000× magnification under 8.0 kV.

### 2.8. Three-Phase Contact Angle

The contact angles of zein and ZCP nanoparticles were measured using an optical contact angle meter (HARKE-SPCAX1, Beijing, China) according to the method described by Cui et al. [22]. Specifically, a 2 mg/mL solution of zein and ZCP nanoparticles was prepared, and 50 μL of the sample solution was dispensed onto a clean silicon wafer and dried at room temperature. A water droplet (3 μL) was slowly dispensed onto the sample surface using a syringe. Images were captured and fitted to determine the contact angle of the sample.

### 2.9. Emulsification Activity Index (EAI) and Emulsification Stability Index (ESI)

The emulsions were prepared as described by Wang et al. [23] with minor modifications, whereas the EAI was calculated according to the turbidimetric definition of Pearce and Kinsella [24]. Briefly, 6 mL of corn oil was mixed with 4 mL of the composite particle dispersion at 60 °C, and the mixture was sheared at 13,000 rpm for 2 min to prepare the emulsion. At room temperature, 50 μL of the freshly prepared emulsion was sampled from the bottom after standing for 10 min and diluted with 5 mL of 0.1% (*w*/*v*) SDS, and absorbance was measured at a wavelength of 500 nm using a UV spectrophotometer (UV-2800A, Unico Instrument Co., Ltd., Shanghai, China). The EAI and ESI were calculated using the formulas shown in (1) and (2):

(1) $$EAI(m^{2}/g)=\frac{2\times 2.303\times A_{0}\times D}{C\times (1-\varphi )\times \theta }$$

(2) $$ESI\left(min\right)=\frac{A_{0}}{A_{0}-A_{10}}\times 10$$

Here, A_0_ is the absorbance of the emulsion at 0 min; A_10_ is the absorbance of the emulsion after standing for 10 min; D is the dilution factor; C is the protein concentration (g/mL); θ is the optical path (0.01 m); and φ is the volume fraction of the oil phase.

### 2.10. Turbiscan Stability Index (TSI)

The Pickering emulsion was prepared using the method described in Section 2.9. The samples were scanned for 12 h using a Turbiscan stability analyzer (Formulaction, Toulouse, France), with slight modifications based on the method described by Wu et al. [25]. By exploiting the differences in the refractive indices of the scattered light, changes in the internal state of the sample over time were detected. The Turbiscan Stability Index (TSI) was calculated using Turbiscan Lab software (version 2.0.0.9) to characterize the emulsion’s stability. Scanning was conducted for 12 h at 25 °C, with scans taken every 5 min.

### 2.11. Data Analysis

All experiments were conducted at least three times. Data are reported as the mean ± standard deviation. Data analysis was performed using SPSS 27 software, with Duncan’s multiple range test applied. The confidence interval was set at 95% (*p* < 0.05). Graphs were generated using Origin 2021 software.

## 3. Results and Discussion

### 3.1. Particle Size of Composite Particles

The particle size and polydispersity index (PDI) are critical indicators for evaluating the colloidal dispersibility and physical stability of nanoparticles. A larger particle size typically signifies a certain degree of particulate aggregation, which can lead to rapid sedimentation under gravity, thereby compromising system stability. Concurrently, the dimensions of the particles dictate their interfacial attachment energy, subsequently governing the droplet aggregation and phase separation behavior of the resulting Pickering emulsions at the macroscopic level. Theoretically, a reduction in particle size accelerates the Brownian diffusion coefficient of the nanoparticles, facilitating their rapid migration and adsorption onto the oil–water interface to assemble a denser interfacial film, which is highly conducive to fabricating emulsions with enhanced stability.

**Figure 1:**
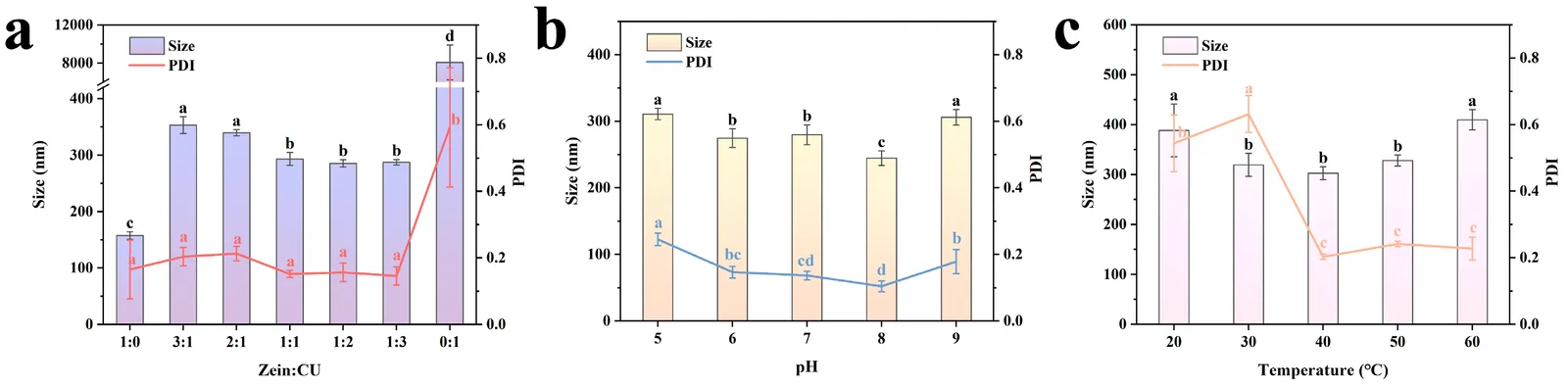
Particle size of composite particles prepared under different conditions: (**a**) zein:CU mass ratio series at pH = 6.0 and T = 40 °C; (**b**) pH series at zein:CU = 1:3 and T = 40 °C; and (**c**) temperature series at zein:CU = 1:3 and pH = 6.0. Lowercase letters represent significant differences between different samples at the *p* < 0.05 level.

Figure 1 illustrates the particle size and PDI of the composite nanoparticles fabricated under different preparation conditions. In the mass ratio series (Figure 1a), pristine zein nanoparticles (zein:CU = 1:0) exhibited an average particle size of 157.53 ± 6.83 nm. After CU incorporation, the hydrodynamic diameter increased compared with pristine zein, indicating the formation of zein–CU composite particles rather than isolated zein nanoparticles [26]. As the CU proportion increased, the particle size decreased from 353.13 ± 14.78 nm (zein:CU = 3:1) to 287.30 ± 4.90 nm (zein:CU = 1:3). This formulation-dependent decrease was accompanied by changes in zeta potential and surface hydrophobicity, suggesting that CU association altered interparticle association and the surface environment of zein particles [6,27]. The PDI values remained below 0.2 across the investigated ratios, indicating a relatively narrow size distribution under these preparation conditions.

As illustrated in Figure 1b, the particle size of the ZCPs first decreased and then increased as the preparation pH increased, reaching a minimum value of 244.63 ± 10.95 nm at pH 8.0. This trend can be related to the pH-dependent ionization of zein. As the pH moved away from the isoelectric region of zein, the deprotonation of amino groups and ionization of carboxyl groups increased the negative surface potential, which enhanced electrostatic repulsion between particles and reduced aggregation. When the pH further increased to 9.0, the particle size increased to 305.93 ± 11.61 nm, probably due to changes in protein conformation and enhanced hydrophobic association under alkaline conditions. The PDI followed a similar trend while remaining at a relatively low level, indicating that the composite dispersions maintained a comparatively uniform size distribution over the pH range examined.

Figure 1c shows the effect of preparation temperature on the particle size of ZCPs. The particle size decreased as the temperature increased to 40 °C and subsequently increased at higher temperatures, with the minimum value of 302.53 ± 13.06 nm observed at 40 °C. Moderate heating can influence zein conformation and protein association in ethanol–water systems, thereby affecting the balance between particle compaction and aggregation [28]. In the present system, the decrease in particle size up to 40 °C was consistent with improved zein–CU association and reduced particle aggregation. At higher temperatures, the increased exposure of hydrophobic domains may promote interparticle association, leading to larger apparent hydrodynamic diameters and higher PDI values.

### 3.2. Zeta Potential

In the continuous phase, the zeta potential dictates the secondary agglomeration tendency and effective particle number density of particulate dispersions by modulating interparticle electrostatic repulsion and electrical double-layer thickness [29]. Fundamentally, the zeta potential reflects the surface charge characteristics of colloidal particles, with its magnitude directly correlating to the intensity of the repulsive electrostatic forces operating between them. Generally, a higher absolute zeta potential value signifies a more pronounced electrostatic repulsion among adjacent entities, which can kinetically suppress droplet coalescence following Brownian collisions, thereby profoundly enhancing the thermodynamic and kinetic stability of the colloidal system [30].

**Figure 2:**
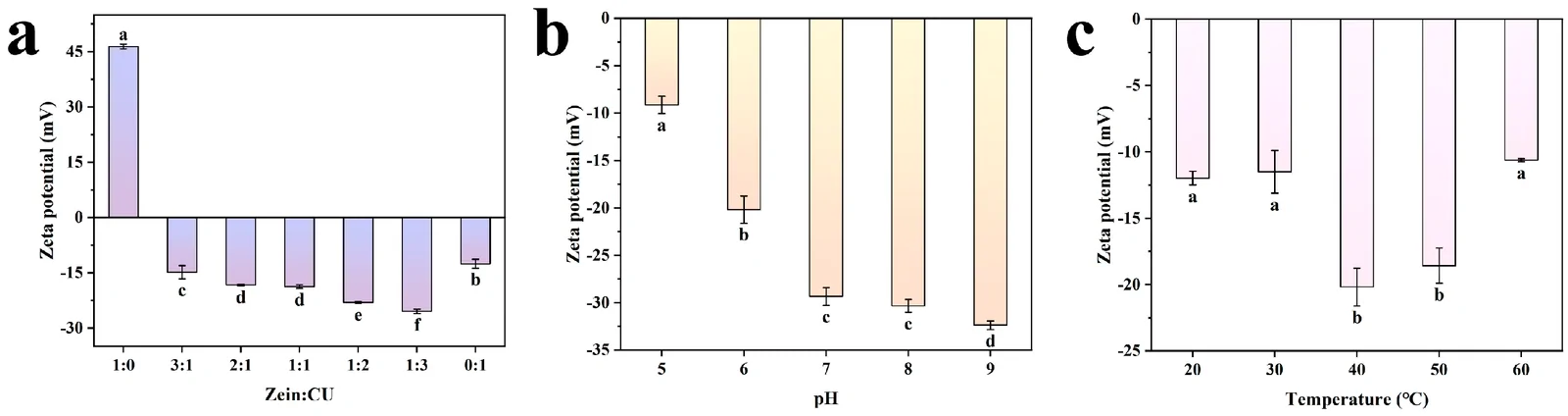
Zeta potential of ZCPs prepared under different conditions: (**a**) zein:CU mass ratio series at pH = 6.0 and T = 40 °C; (**b**) pH series at zein:CU = 1:3 and T = 40 °C; and (**c**) temperature series at zein:CU = 1:3 and pH = 6.0. Lowercase letters represent significant differences between different samples at the *p* < 0.05 level.

Figure 2 presents the zeta potential of the composite nanoparticles fabricated under different preparation conditions. In the mass ratio series (Figure 2a), pristine zein showed a positive zeta potential of +46.37 mV at pH 6.0, which is consistent with the protonation of amino acid residues when the system pH is close to or below the isoelectric region of zein [31]. The incorporation of CU shifted the zeta potential of ZCPs toward negative values, reaching −25.45 mV at a zein:CU mass ratio of 1:3. This progressive shift indicates that CU incorporation markedly altered the electrokinetic characteristics and surface environment of the composite particles. Together with the particle size results, the increased absolute zeta potential at higher CU proportions suggests enhanced electrostatic repulsion between composite particles and improved colloidal dispersibility. Parallelly, Jin et al. [32] demonstrated that the complexation of positively charged proteins with neutral polysaccharides substantially shifted the zeta potential landscape, thereby optimizing the electrostatic and steric stability of the resulting emulsions.

The response of zeta potential to varying pH levels is presented in Figure 2b. As the pH increased from 5.0 to 9.0, the zeta potential of the ZCPs decreased from −9.13 ± 0.92 mV to −32.34 ± 0.47 mV. This trend was mainly associated with the pH-dependent ionization of zein. With increasing pH, protonated amino groups are progressively deprotonated, while carboxyl groups become negatively charged carboxylate groups, thereby increasing the negative surface potential of the particles. The greater absolute zeta potential at alkaline pH values indicates stronger interparticle electrostatic repulsion, which was consistent with the smaller particle size observed at pH 8.0 and the improved emulsifying behavior discussed below.

The zeta potential results for the composite particles prepared at different temperatures are shown in Figure 2c. As the temperature increased, the zeta potential first decreased in magnitude and then increased again, reaching the most negative value of −21.18 ± 1.43 mV at 40 °C before increasing to −10.63 ± 0.12 mV at 60 °C. This trend was consistent with the particle size results, suggesting that preparation temperature affected both particle association and the surface characteristics of ZCPs. The greater absolute zeta potential at 40 °C may contribute to stronger electrostatic repulsion between particles, whereas the lower magnitude at higher temperatures may favor aggregation.

### 3.3. Microstructure

**Figure 3:**
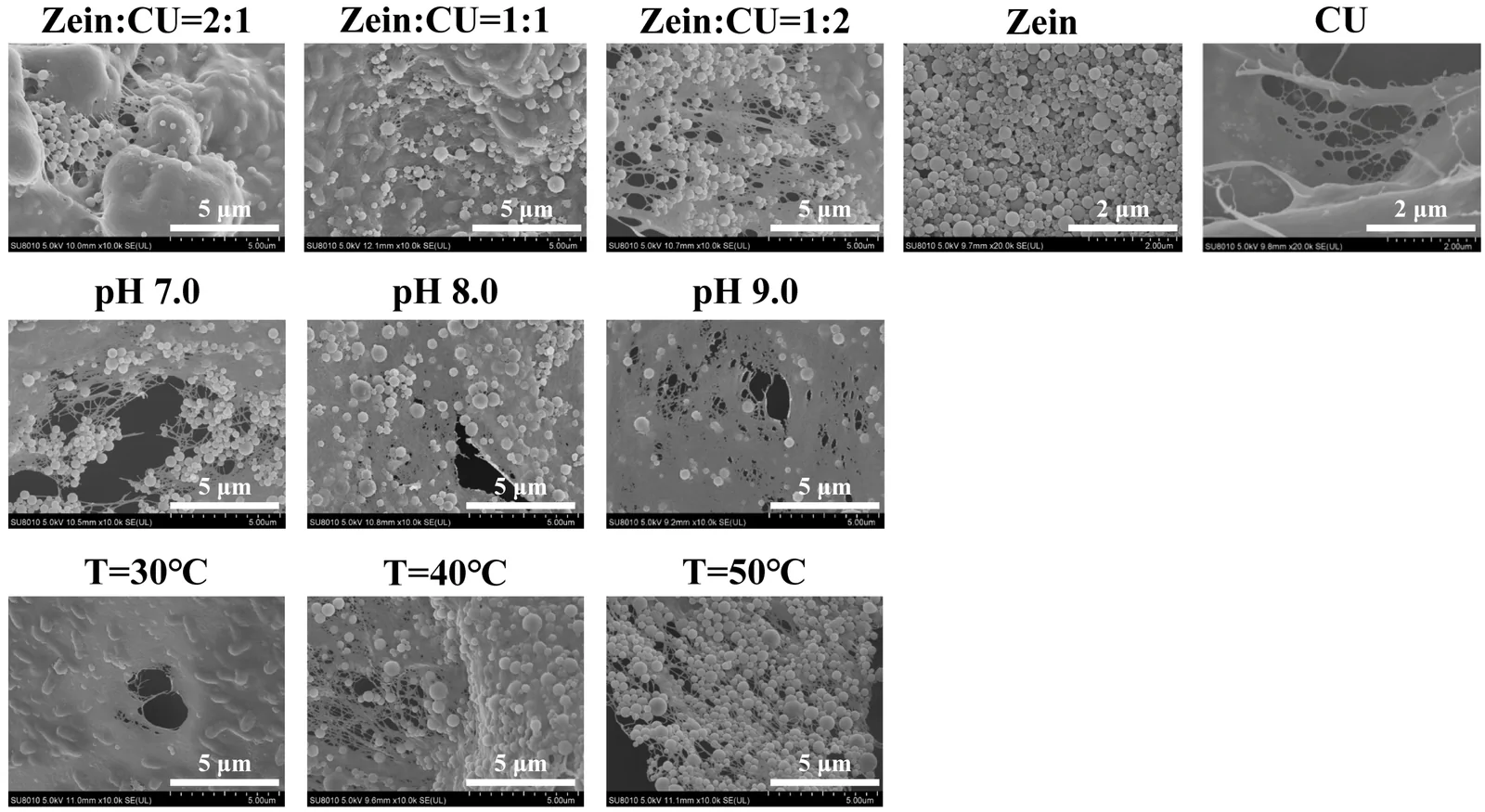
SEM images of ZCPs prepared under different conditions: zein:CU mass ratio series at pH = 6.0 and T = 40 °C; pH series at zein:CU = 1:3 and T = 40 °C; and temperature series at zein:CU = 1:3 and pH = 6.0.

To visually elucidate the micro-morphological evolution of the composite nanoparticles, three representative samples (zein:CU = 2:1, 1:1, and 1:2, *w*/*w*) were selected based on the dynamic light scattering (DLS) results for SEM observations. As manifested in Figure 3, pristine zein assembled into spherical nanoparticles characterized by well-defined boundaries and uniform dimensions during the anti-solvent precipitation process, whereas pure curdlan (CU) exhibited a smooth-surfaced, three-dimensional reticular fibrous sheet architecture. The incorporation of CU produced composition-dependent changes in the surface morphology and aggregation state of the dried samples. At zein:CU = 2:1, protein-rich aggregates were more evident, consistent with the larger hydrodynamic diameter observed by DLS. At zein:CU = 1:1 and 1:2, the dried composite particles showed a more heterogeneous and rougher surface morphology, indicating that CU association changed the organization of zein particles during particle formation and drying [33]. These morphological differences were consistent with the changes in particle size, zeta potential, and surface hydrophobicity.

To evaluate the morphic variations, three typical samples fabricated under varying pH levels (pH 7.0, 8.0, and 9.0) were selected for SEM examination. The micrographs revealed that with escalating pH, the surface topography of the composite nanoparticles transitioned from a highly wrinkled and rough profile to a smooth texture, accompanied by a noticeable mitigation in the degree of particulate clustering. This structural evolution was fundamentally governed by the attenuation of the intense intermolecular hydrophobic associations among the protein chains. Specifically, the higher pH amplified the surface negative charge density of the biopolymers; the resulting fierce electrostatic repulsion effectively hindered the orderly alignment and hydrophobic association of the protein matrices. This electrical barrier underscores that an elevated pH significantly reinforces the anti-aggregation stability of the composite nanoparticles. Concomitantly, the dimensions of the self-assembled complex shrunk progressively with expanding pH, yielding a more monodisperse and uniform system—a behavioral pattern in excellent alignment with the observations documented by Ge et al. [34] regarding zein–carrageenan complexes.

Representative samples prepared at 30 °C, 40 °C, and 50 °C were selected to examine temperature-dependent morphological changes. The dried samples prepared at 30 °C showed a relatively smooth and continuous morphology with visible particle domains. At 40 °C, the samples exhibited a rougher and more dispersed particle organization, consistent with the smaller particle size and higher absolute zeta potential measured under this condition. At 50 °C, more pronounced heterogeneity and particle association were observed. These results indicate that preparation temperature affected the dried morphology and aggregation state of ZCPs, which was consistent with the temperature-dependent changes in colloidal properties and emulsifying performance.

### 3.4. FTIR

**Figure 4:**
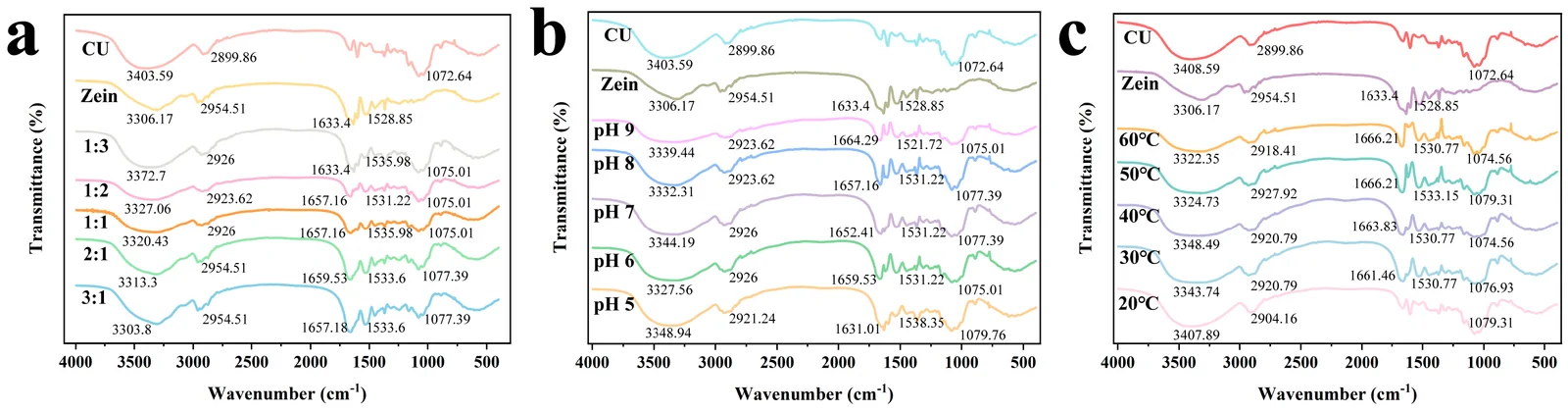
FTIR spectra of ZCPs prepared under different conditions: (**a**) zein:CU mass ratio series at pH = 6.0 and T = 40 °C; (**b**) pH series at zein:CU = 1:3 and T = 40 °C; and (**c**) temperature series at zein:CU = 1:3 and pH = 6.0.

FTIR spectroscopic analysis was performed to clarify the variations in functional groups and decipher the underlying intermolecular interactions governing the biopolymer assembly. As illustrated in Figure 4a, pristine CU exhibited a strong and broad stretching vibration peak of hydroxyl groups (-OH) at 3403.59 cm^−1^, which originates from the abundant hydrophilic moieties along its polysaccharide backbone. Conversely, pure zein displayed a characteristic absorption band at 3306.17 cm^−1^, corresponding to the -NH or -OH stretching vibrations. In the ZCP composite systems, the -OH band of CU shifted progressively toward lower wavenumbers. This redshift suggests changes in the hydrogen-bonding environment following the association of zein with CU [35]. This observation aligns with the findings documented by Zeng et al. [19] for zein–sodium alginate complexes, where the redshift within the 3600–3200 cm^−1^ region was attributed to the spatial reorganization of the hydrogen-bonding topology of the protein. For pristine zein, the prominent peak at 1633.40 cm^−1^ is assigned to the amide I band (predominantly C-O stretching vibration). In the ZCP complexes, this band experienced a noticeable redshift, suggesting that the newly formed hydrogen bonds and hydrophobic associations disrupted the native α-helix conformation of zein, thereby driving a secondary structural transition toward a more thermodynamically stable β-sheet architecture [36]. Concurrently, the absorption peak at 1528.85 cm^−1^, corresponding to the amide II band (N-H bending and C-N stretching), migrated toward higher wavenumbers in the complexes. The shift in the amide II band further indicates changes in the local molecular environment of zein following CU incorporation [37], which highly mirrors the results of the zeta potential analysis and echoes the observations reported by Zhang et al. [38] on soy protein–polysaccharide complexes. Regarding the carbohydrate fingerprints, the peaks at 1072 cm^−1^ (glycosidic bond stretching) and 898 cm^−1^ (β-1,3-glucosidic linkage) increased in intensity with the expanding CU mass ratio. This retention confirms that the primary backbone configuration of CU remained intact during self-assembly, enabling its polymeric network to serve as a supportive physical skeleton for the hybrid matrix. Comparative analysis revealed that at a zein-to-CU mass ratio of 2:1, the displacement of the amide I band reached its maximum; at a 1:1 ratio, a superior thermodynamic equilibrium between hydrogen bonding and hydrophobic association was achieved, culminating in a highly integrated and structurally stable composite system.

The FTIR spectra of composite particles prepared at different pH values are shown in Figure 4b. As the pH increased from 5.0 to 9.0, the band in the 3200–3500 cm^−1^ region shifted and broadened, indicating that pH affected the hydrogen-bonding environment and local molecular organization of the composite particles. The changes in the amide I and amide II regions further suggested that the ionization state of zein influenced protein conformation and zein–CU association. These spectral variations were consistent with the pH-dependent changes in particle size and zeta potential, indicating that pH regulated the colloidal state of ZCPs through combined effects on protein charge, molecular association, and particle aggregation.

As shown in Figure 4c, the characteristic bands in the 3200–3400 cm^−1^ region shifted with increasing preparation temperature, with more pronounced changes observed around 40 °C, indicating a temperature-dependent alteration in the hydrogen-bonding environment within the composite system. The C–H stretching band near 2954.51 cm^−1^ also shifted, suggesting changes in the local hydrophobic environment and molecular packing. Simultaneously, shifts in the amide I and amide II bands indicated that preparation temperature altered the local conformational environment of zein, suggesting a temperature-dependent reorganization of protein–polysaccharide associations within the composite particles. These spectral variations were consistent with the corresponding changes in particle size, zeta potential, and surface hydrophobicity, indicating that temperature influenced the molecular organization and colloidal state of the zein–CU composite particles.

### 3.5. Surface Hydrophobicity

**Figure 5:**
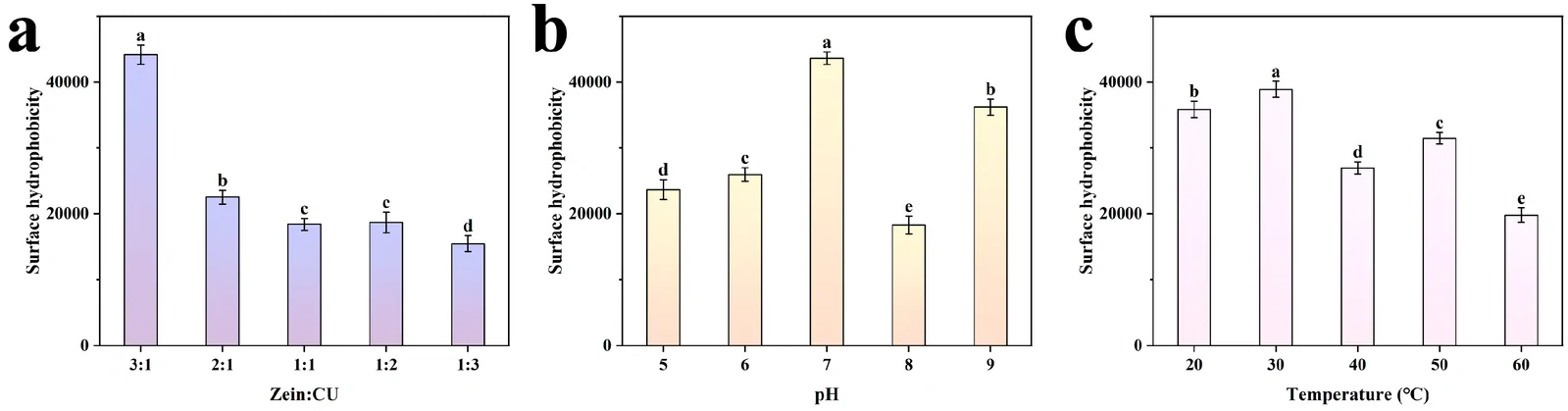
Surface hydrophobicity of ZCPs prepared under different conditions: (**a**) zein:CU mass ratio series at pH = 6.0 and T = 40 °C; (**b**) pH series at zein:CU = 1:3 and T = 40 °C; and (**c**) temperature series at zein:CU = 1:3 and pH = 6.0. Lowercase letters represent significant differences between different samples at the *p* < 0.05 level.

Surface hydrophobicity (H_0_) is closely related to protein conformation, exposed hydrophobic domains, and interfacial adsorption behavior [39,40]. ANS was used as a fluorescent probe to evaluate the accessible hydrophobic regions on ZCP surfaces. As shown in Figure 5a, pristine zein exhibited high surface hydrophobicity, reflecting the hydrophobic character of zein particles. In the mass ratio series, increasing the CU proportion progressively decreased H_0_. This decrease suggests that CU association reduced the accessibility of hydrophobic zein domains, producing a more hydrated particle surface. The reduction in surface hydrophobicity was consistent with the zeta potential shift and the change in contact angle, indicating that CU incorporation modified the surface characteristics of zein particles.

The H_0_ values of the composite nanoparticles fabricated under different pH levels are presented in Figure 5b. ZCPs exhibited the highest H_0_ at pH 7.0, which may be related to increased protein association near the isoelectric region of zein and greater exposure of hydrophobic domains. At pH 8.0, H_0_ decreased, consistent with the higher absolute zeta potential and smaller particle size observed under this condition. The increase in negative surface potential may have reduced aggregation and changed the accessibility of hydrophobic regions. When the pH increased to 9.0, the rebound in H_0_ may be associated with alkaline-induced changes in protein conformation and exposure of hydrophobic domains [41].

The effect of preparation temperature on H_0_ is illustrated in Figure 5c. The H_0_ values decreased as the temperature increased, indicating that temperature altered the accessibility of hydrophobic domains on the particle surface. The decrease up to 40 °C was consistent with reduced particle size and improved electrostatic repulsion, suggesting a more balanced zein–CU association and less exposed hydrophobic surface. At higher temperatures, protein association and particle aggregation may reduce the accessible surface area for ANS binding, leading to lower apparent H_0_ values.

### 3.6. XRD

**Figure 6:**
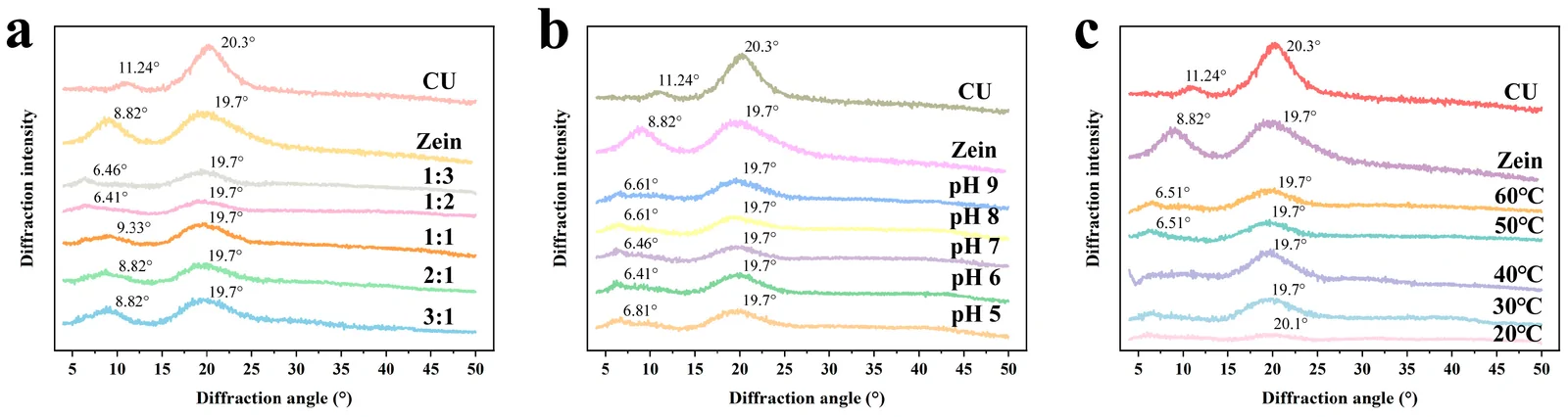
XRD patterns of ZCPs prepared under different conditions: (**a**) zein:CU mass ratio series at pH = 6.0 and T = 40 °C; (**b**) pH series at zein:CU = 1:3 and T = 40 °C; and (**c**) temperature series at zein:CU = 1:3 and pH = 6.0.

X-ray diffraction was used to evaluate changes in molecular order during zein–CU composite particle formation. As shown in Figure 6a, zein exhibited broad diffraction peaks at 2θ ≈ 8.82° and 19.70°, reflecting its mainly amorphous character with short-range molecular order [42]. CU exhibited characteristic peaks at 2θ ≈ 11.24° and 20.30°, which are related to ordered polysaccharide chain organization. The composite systems showed broad diffraction patterns without obvious new crystalline peaks. The weakened intensity and broadening of the main peaks in the ZCPs indicate reduced molecular order and increased amorphous character after zein–CU association.

The XRD patterns of samples prepared at different pH values are shown in Figure 6b. Compared with the native components, the characteristic CU peak at 11.24° weakened or disappeared in the composite particles, and the zein-related peak at 8.82° also became less pronounced at pH 7.0–9.0. These changes indicate that pH affected the short-range organization of the protein–polysaccharide system and promoted a more amorphous composite structure. A weak low-angle signal was observed in the composite samples; however, the exact peak position and assignment require verification from the original XRD data, particularly regarding whether the signal appears at 2θ = 6.6° or 6.8°.

The XRD patterns of composite particles prepared at different temperatures are shown in Figure 6c. As the preparation temperature increased, the characteristic CU peak at 20.30° weakened or disappeared, while the broad zein-related peak near 19.70° remained but showed reduced intensity and increased width. The disappearance or weakening of peaks at 8.82° and 11.24° further indicated reduced short-range order and increased amorphous character. These results were consistent with the FTIR spectra, suggesting that preparation temperature changed the local molecular organization of ZCPs.

### 3.7. Three-Phase Contact Angle

The wettability of solid particles is a pivotal determinant for evaluating their particulate emulsifying efficiency and the long-term stabilization kinetics of the resulting Pickering emulsions. It not only dictates the static thermodynamic positioning of the particles at the oil–water interface but also unveils the fundamental mechanical barrier that suppresses droplet coalescence [43]. Typically, plant proteins exhibit soft-particle characteristics, which undergo pronounced conformational deformation and structural spreading upon adsorption at the fluid–fluid interface. Furthermore, the characterization of particulate wettability directly dictates the preferred interfacial curvature around the liquid droplets. Thermodynamically, when the three-phase contact angle is less than 90°, the particles are preferentially wetted by water, and their bulk spatial partitioning remains predominantly within the continuous aqueous phase. This spatial distribution forces the fluid interface to curve toward the organic phase, thereby favoring the formulation of O/W Pickering emulsions. Conversely, when θ > 90°, the particulate matrix partition is primarily swept into the oil phase, inducing an opposite curvature that thermodynamically biases the system toward creating W/O emulsions [2].

**Figure 7:**
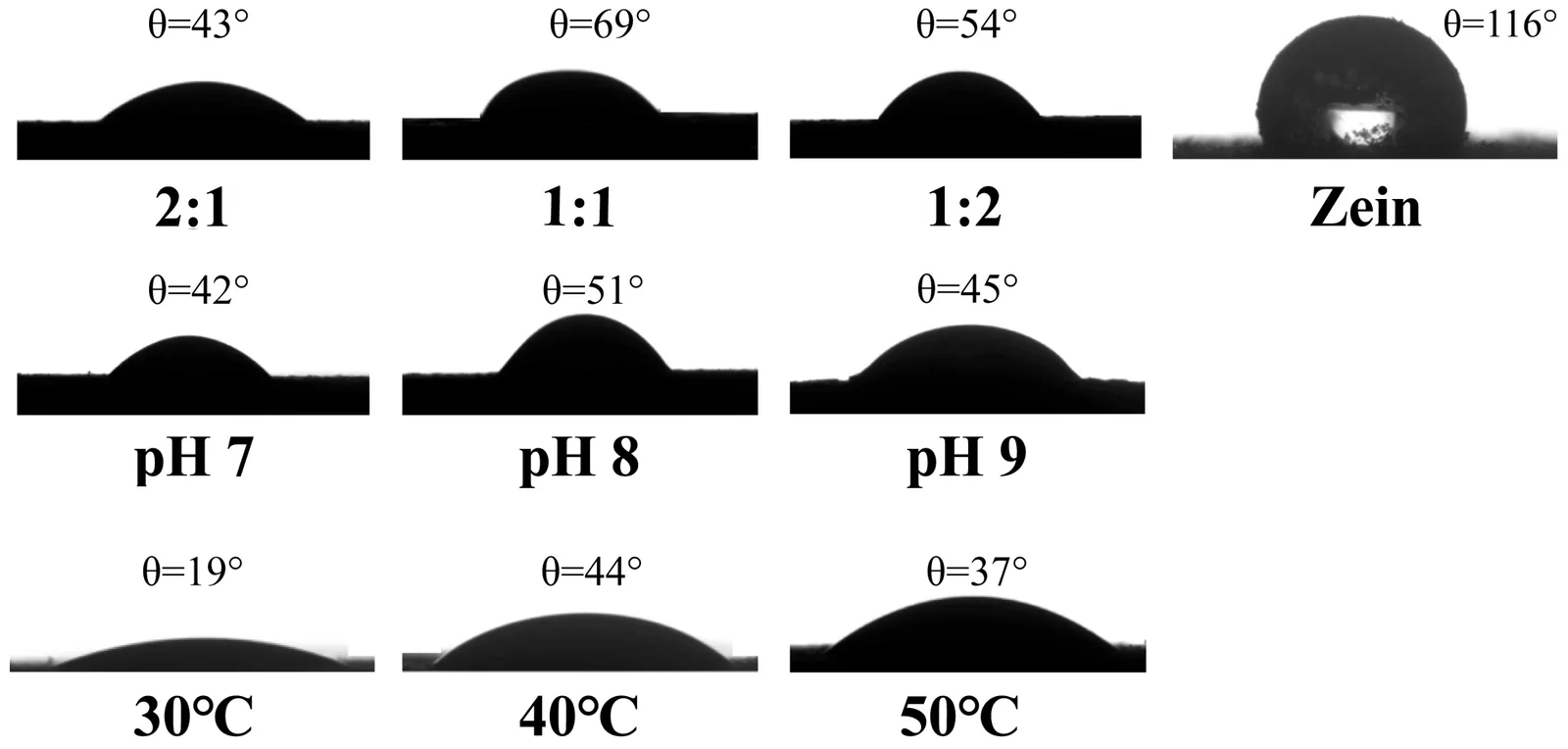
Three-phase contact angles of ZCPs prepared under different conditions: zein:CU mass ratio series at pH = 6.0 and T = 40 °C; pH series at zein:CU = 1:3 and T = 40 °C; and temperature series at zein:CU = 1:3 and pH = 6.0.

As shown in Figure 7, the three-phase contact angle of zein was 116°, indicating a relatively hydrophobic particle film. After CU incorporation, the contact angle decreased markedly. At zein:CU mass ratios of 2:1, 1:1, and 1:2, the contact angles were 43°, 69°, and 54°, respectively. The decrease compared with zein suggests that CU association increased the hydrophilicity of the particle film, whereas the higher value at zein:CU = 1:1 indicated a more balanced wettability. This wettability balance may facilitate particle adsorption at the oil–water interface and contribute to improved emulsifying performance within the investigated mass ratio series.

When the preparation pH increased from 7.0 to 8.0, the contact angle increased from 42° to 51° and then decreased to 45° at pH 9.0. The higher value at pH 8.0 indicates a relatively more balanced wettability of the ZCPs, which was consistent with the smaller particle size and stronger electrostatic repulsion under this condition. At pH 9.0, alkaline-induced changes in protein conformation and the higher negative surface potential may have increased the hydrophilic character of the particle surface, leading to a lower contact angle [44].

Under different preparation temperatures, the contact angle first increased and then decreased. The contact angle increased from 19° at 30 °C to 44° at 40 °C and then decreased to 37° at 50 °C. This trend suggests that preparation temperature altered the relative wettability of the ZCPs. The comparatively higher contact angle at 40 °C, together with the smaller particle size and more negative zeta potential, indicates a favorable balance between hydrophilic and hydrophobic surface characteristics among the temperatures examined.

### 3.8. Emulsification Activity Index (EAI) and Emulsification Stability Index (ESI)

**Figure 8:**
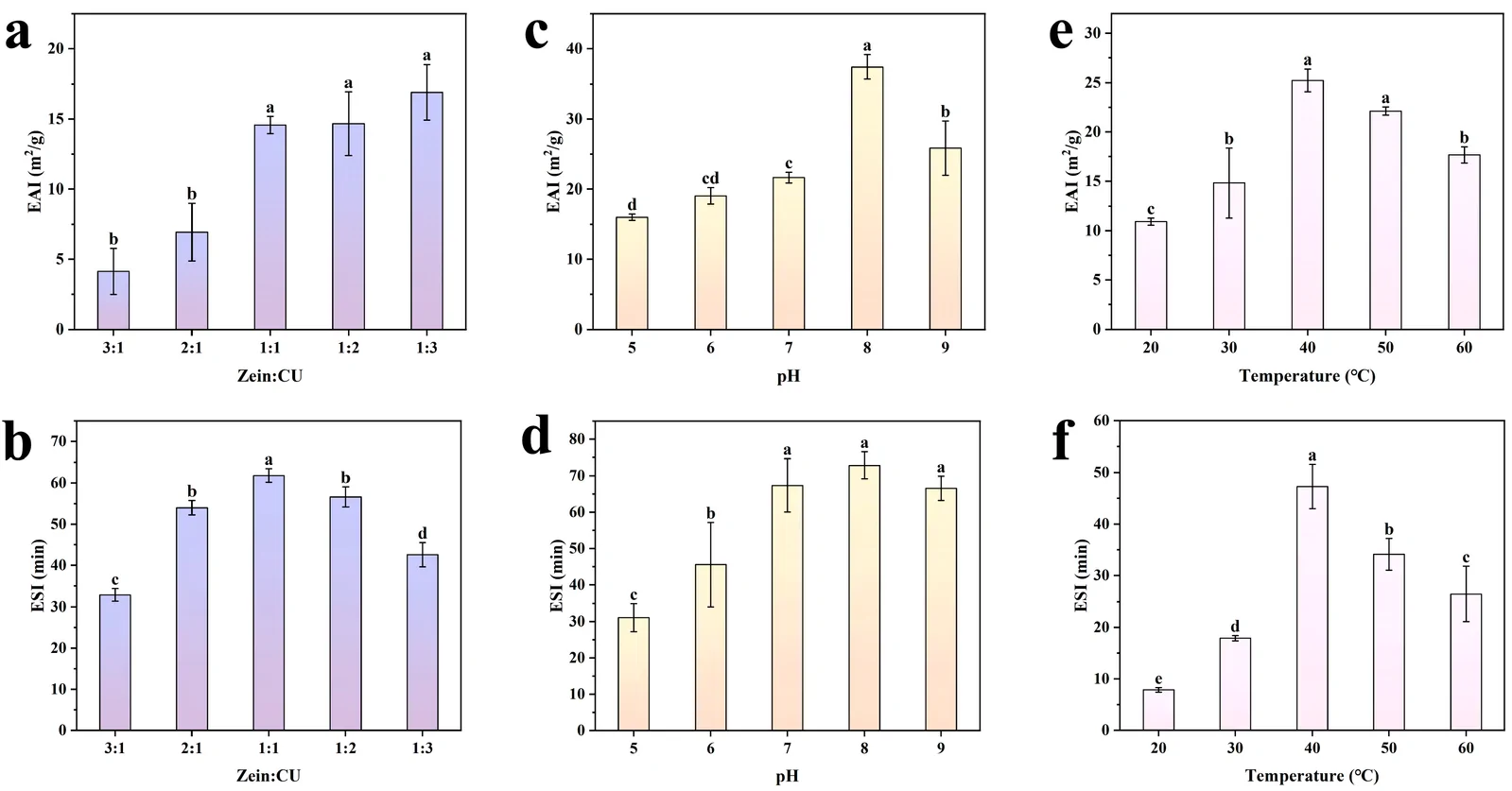
Emulsification activity index of ZCPs prepared under different conditions: (**a**) zein:CU mass ratio series at pH = 6.0 and T = 40 °C; (**c**) pH series at zein:CU = 1:3 and T = 40 °C; and (**e**) temperature series at zein:CU = 1:3 and pH = 6.0. Emulsification stability index of ZCPs prepared under same conditions: (**b**) mass ratio series, (**d**) pH series, and (**f**) temperature series. Lowercase letters represent significant differences between different samples at the *p* < 0.05 level.

The EAI measures the efficiency of proteins and polysaccharides to quickly adsorb to the oil–water interface and form an emulsion layer, and the ESI mainly evaluates the resistance of emulsion droplets to aggregation and flocculation. The EAI results under different mass ratios are shown in Figure 8a. With increasing CU proportion, emulsifying activity increased, indicating that CU-associated changes in particle size, surface charge, and wettability favored interfacial adsorption during homogenization. As shown in Figure 8b, the ESI first increased and then decreased with increasing CU proportion. The poorer stability at zein:CU = 3:1 may be related to larger particle size and insufficient steric/electrostatic stabilization [45], whereas excessive CU at zein:CU = 1:3 may promote droplet flocculation or reduce effective particle adsorption. The zein:CU = 1:1 sample showed the most favorable stability within the investigated mass ratio series, consistent with its balanced wettability and moderate particle surface properties.

The EAI of composite particles prepared at different pH values is shown in Figure 8c. The EAI first increased and then decreased, reaching a maximum of 37.44 ± 1.73 m^2^/g at pH 8.0. This may be attributed to the smaller particle size and suitable wettability at this pH, which can provide more particles to cover the newly formed oil–water interface. As shown in Figure 8d, the ESI increased with increasing pH. The stronger negative zeta potential at alkaline pH likely enhanced electrostatic repulsion between particles and droplets, thereby reducing aggregation and coalescence during the stability test. These results indicate that pH-dependent changes in particle size, surface potential, and wettability collectively affected emulsifying behavior.

The emulsifying activity and stability of composite particles prepared at different temperatures are shown in Figure 8e,f. Both the EAI and ESI first increased and then decreased with increasing temperature, reaching maximum values at 40 °C, with an EAI of 25.23 ± 1.16 m^2^/g and an ESI of 47.28 ± 4.31 min. This behavior was consistent with the temperature-dependent changes in particle size, zeta potential, surface hydrophobicity, and static water contact angle. Particles prepared at 40 °C showed a favorable balance between colloidal dispersibility and surface wettability, which likely promoted interfacial adsorption and improved resistance to droplet aggregation. At higher temperatures, increased particle association and lower absolute zeta potential may reduce interfacial coverage efficiency, leading to a decreased EAI and ESI.

### 3.9. TSI

The Turbiscan analyzer can monitor the backscattering light intensity variations (ΔBS) in samples in real time and in situ under non-destructive conditions. The smaller the ΔBS, the stronger the stability of the Pickering emulsion. When structural changes such as creaming or flocculation occur between droplets, the morphology and intensity of the backscattering curve change locally or globally [46]. When the Pickering emulsion undergoes creaming and phase separation, the BS curves show opposite systematic changes at the top and bottom, presenting a gradual advancement feature along the height, reflecting the migration and concentration of droplets under the gravitational field. The variation speed is related to the droplet size and the structural network in the continuous phase. Comparing TSI values under the same conditions can help analyze the stability differences in samples. The smaller the curve slope and TSI value, the higher the stability of the system [47]. Overall, the TSI curve can not only be used to evaluate the stability differences between different samples but also provide macroscopic evidence for the dominant destabilization mechanism of Pickering emulsions during the 12 h monitoring period.

**Figure 9:**
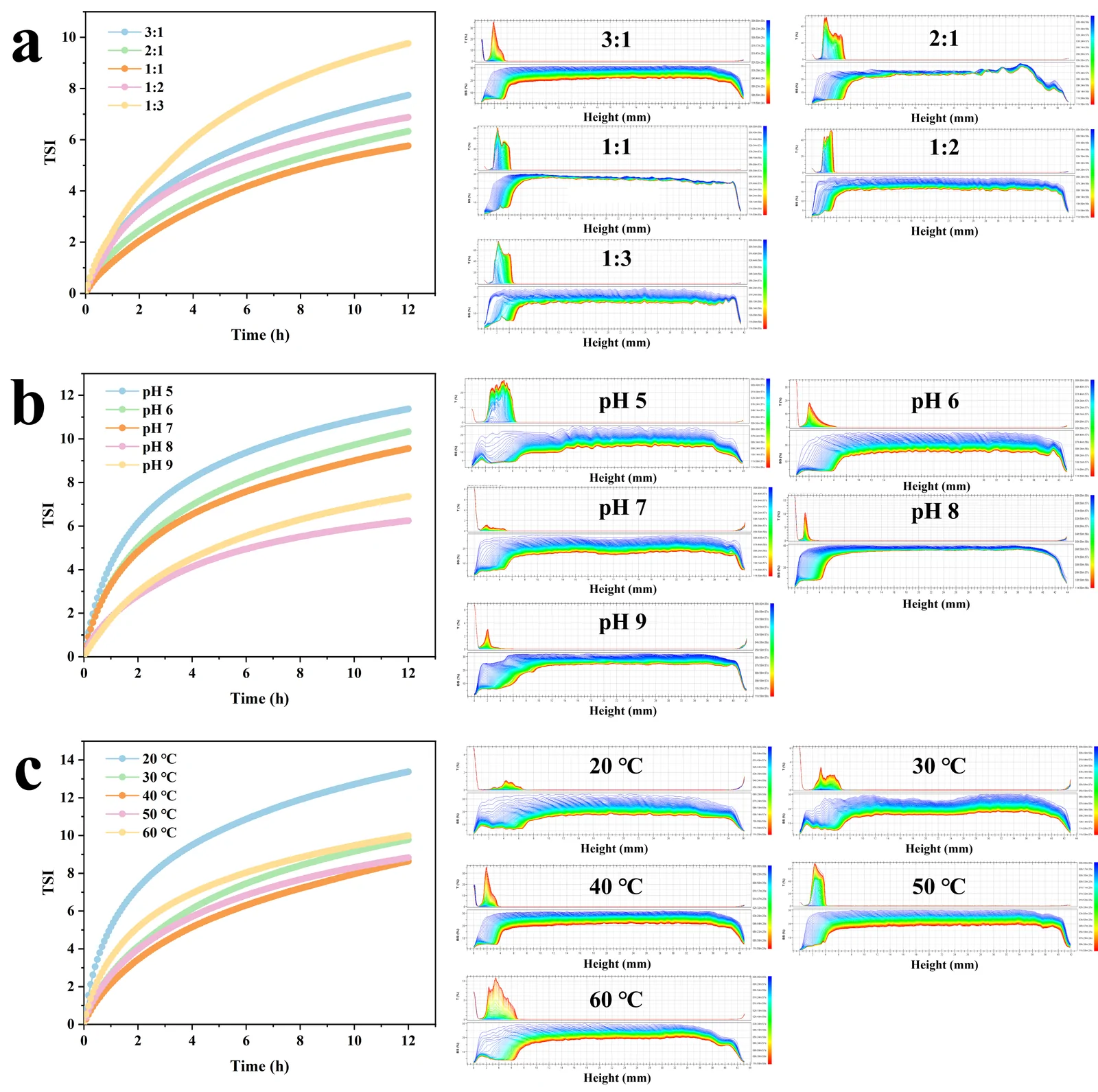
TSI results for Pickering emulsions stabilized by ZCPs prepared under different conditions: (**a**) zein:CU mass ratio series at pH = 6.0 and T = 40 °C; (**b**) pH series at zein:CU = 1:3 and T = 40 °C; and (**c**) temperature series at zein:CU = 1:3 and pH = 6.0.

The stability results of Pickering emulsions prepared with different zein:CU mass ratios are shown in Figure 9a. After 12 h, the zein:CU = 1:1 sample showed the lowest TSI value (5.76), followed by the 2:1, 1:2, 3:1, and 1:3 samples, with TSI values of 6.32, 6.87, 7.73, and 9.76, respectively. This trend was consistent with the ESI results, confirming that the 1:1 formulation had the best physical stability during the observation period within the mass ratio series. The BS profiles indicated changes at the bottom, middle, and top of the sample, corresponding mainly to clarification, droplet migration, and aggregation/flocculation. Larger changes for the 3:1 and 1:3 samples suggest greater early-stage destabilization, whereas the higher overlap of BS curves for the 1:1 sample indicates a more stable droplet distribution.

The stability results of Pickering emulsions prepared at different pH values are shown in Figure 9b. The lowest TSI was observed at pH 8.0, followed by pHs 9.0, 7.0, 6.0, and 5.0, with TSI values of 6.25, 7.35, 9.56, 10.33, and 11.37, respectively, after 12 h. This trend was consistent with the EAI and ESI results. The smaller particle size, stronger negative zeta potential, and suitable wettability at pH 8.0 likely promoted more effective interfacial stabilization. The BS profiles showed that destabilization mainly occurred during the early stage, with changes at the bottom and top regions reflecting clarification and droplet migration and changes in the middle region reflecting aggregation or flocculation.

As shown in Figure 9c, the TSI of emulsions prepared with particles fabricated at different temperatures first decreased and then increased, reaching the lowest value at 40 °C. The TSI decreased from 13.37 to 8.63 as temperature increased to 40 °C and then increased to 9.99 at higher temperatures. The lower TSI at 40 °C was consistent with the higher EAI and ESI values, indicating better short-term physical stability. The corresponding BS profiles showed slower changes in the bottom and middle regions for the 40 °C emulsion, suggesting reduced droplet migration and flocculation compared with the other temperature treatments.

## 4. Conclusions

This study systematically investigated the regulatory laws of preparation processes (mass ratio, reaction pH, and reaction temperature) on the self-assembled structure changes in zein–CU composite nanoparticles and their capacity to stabilize Pickering emulsions. FTIR, XRD, and SEM analyses, together with particle size, zeta potential, and surface hydrophobicity measurements, indicated the formation of zein–CU composite particles accompanied by changes in their molecular organization and surface characteristics. Within the investigated mass ratio series, zein:CU = 1:1 showed the most favorable emulsion stability during the observation period. Among the pH conditions examined, pH 8.0 produced smaller particles with a higher absolute zeta potential and improved emulsifying behavior. Among the temperatures examined, 40 °C produced particles with a favorable balance of colloidal dispersibility and surface wettability, resulting in the higher EAI, ESI, and short-term physical stability of the corresponding Pickering emulsions. These findings clarify the relationship between fabrication conditions, particle properties, and emulsifying performance, supporting the potential application of zein–CU composite nanoparticles as food-grade particulate stabilizers.

## Data Availability

The original contributions presented in the study are included in the article, further inquiries can be directed to the corresponding author.

## Abbreviations

The following abbreviations are used in this manuscript:

CU	Curdlan
ZCPs	Zein–curdlan composite particles
SEM	Scanning electron microscope
XRD	X-ray diffraction
EAI	Emulsification activity index
ESI	Emulsification stability index
ANS	8-phenyl-1-naphthalenesulfonic acid
H_0_	Surface hydrophobicity
TSI	Turbiscan stability index
